# Transcript Assembly and Quantification by RNA-Seq Reveals Differentially Expressed Genes between Soft-Endocarp and Hard-Endocarp Hawthorns

**DOI:** 10.1371/journal.pone.0072910

**Published:** 2013-09-05

**Authors:** Hongyan Dai, Guofen Han, Yujiao Yan, Feng Zhang, Zhongchi Liu, Xiaoming Li, Wenran Li, Yue Ma, He Li, Yuexue Liu, Zhihong Zhang

**Affiliations:** 1 College of Horticulture, Shenyang Agricultural University, Shenyang, China; 2 College of Bioscience and Biotechnology, Shenyang Agricultural University, Shenyang, China; 3 Department of Cell Biology and Molecular Genetics, University of Maryland, College Park, Maryland, United States of America; Kyushu Institute of Technology, Japan

## Abstract

Hawthorn (*Crataegus* spp.) is an important pome with a long history as a fruit, an ornamental, and a source of medicine. Fruits of hawthorn are marked by hard stony endocarps, but a hawthorn germplasm with soft and thin endocarp was found in Liaoning province of China. To elucidate the molecular mechanism underlying the soft endocarp of hawthorn, we conducted a *de novo* assembly of the fruit transcriptome of *Crataegus pinnatifida* and compared gene expression profiles between the soft-endocarp and the hard-endocarp hawthorn varieties. *De novo* assembly yielded 52,673 putative unigenes, 20.4% of which are longer than 1,000 bp. Among the high-quality unique sequences, 35,979 (68.3%) had at least one significant match to an existing gene model. A total of 1,218 genes, represented 2.31% total putative unigenes, were differentially expressed between the soft-endocarp hawthorn and the hard-endocarp hawthorn. Among these differentially expressed genes, a number of lignin biosynthetic pathway genes were down-regulated while almost all the flavonoid biosynthetic pathway genes were strongly up-regulated, concomitant with the formation of soft endocarp. In addition, we have identified some MYB and NAC transcription factors that could potentially control lignin and flavonoid biosynthesis. The altered expression levels of the genes encoding lignin biosynthetic enzymes, MYB and NAC transcription factors were confirmed by quantitative RT-PCR. This is the first transcriptome analysis of *Crataegus* genus. The high quality ESTs generated in this study will aid future gene cloning from hawthorn. Our study provides important insights into the molecular mechanisms underlying soft endocarp formation in hawthorn.

## Introduction

Hawthorn (*Crataegus* spp.), a genus of the Rosaceae family, is an important plant that grows in Asia, Europe, North and Central America, and northern South America. It is a small shrub or spreading tree with thorny branches, three-to five-lobed deciduous leaves, white flowers, red, orange, and yellow-green or yellow fruits [1]. Hawthorn is found widely in open woodlands, as well as in hilly montane forests. Numerous hybrids and variants exist in the *Crataegus* genus, and the total number of species is estimated at between 140 and 200 [2]. Hawthorn fruit has been consumed for 2,500 years in China, primarily to improve digestion and decrease food stasis as hawthorn fruit is rich in crataegus acid [3]. The fruit may be eaten raw or processed into sauce, slices, or juice. Besides its wide use as an ornamental, hawthorn is considered one of the oldest pharmaceutical plants, and has been used as an alternative therapy for a variety of cardiovascular conditions [1], [4].

**Figure 1:**
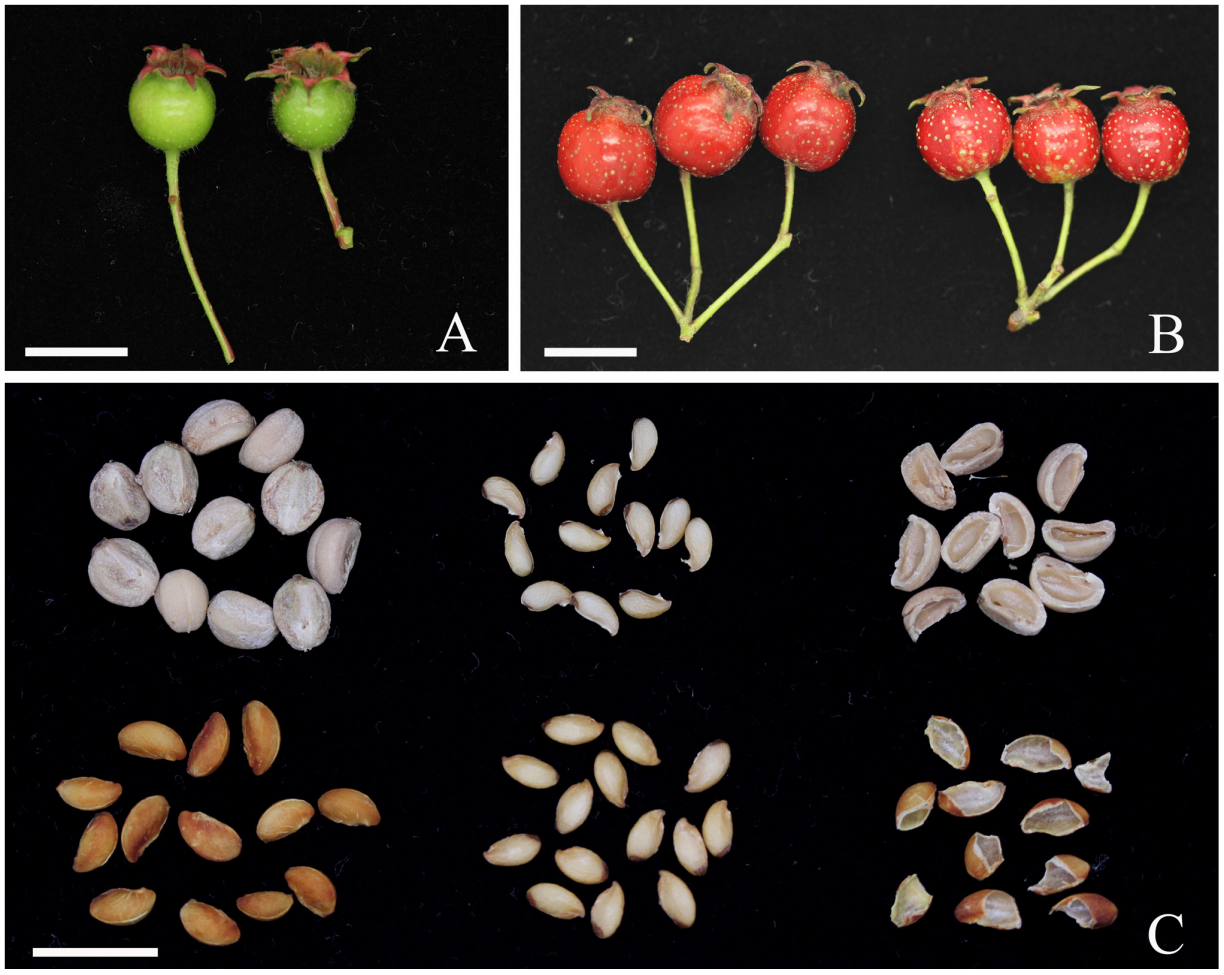
Comparisons between the hard-endocarp hawthorn (H8) and the soft-endocarp hawthorn (S7). (a) Young fruit (23 DAB) of H8 (left) and S7 (right). (b) Ripen fruits (118 DAB) of H8 (left) and S7 (right). (c) Pyrenes (left), seeds (middle) and broken endocarp (right) of H8 (up) and S7 (down). Ba = 1 cm.

In botany, hawthorn is a pome, a type of fruit produced by flowering plants in the Rosaceae family. Fruits of hawthorn are marked by hard stony endocarps that are readily dispersed by larger birds and small rodents [5], [6]. Both the wild germplasm and cultivated varieties of *Crataegus* are abundant in China [7]. ‘Ruanheshanzha’, the soft-endocarp hawthorn, is a special germplasm of *Crataegus pinnatifida,* first found in Liaoning province of China in 1958 by the researchers of Institute of Horticulture, Liaoning Academy of Agricultural Sciences, China [8]. The endocarps of ‘Ruanheshanzha’ are soft, thin (Figure 1), and edible. The seeds can be germinated after one winter stratification. In contrast, the common hawthorn’s seeds with hard wood-like endocarps (pyrenes) usually germinate after 2 to 3 years of stratification [9]. In addition, the true seed (stones containing embryos) of ‘Ruanheshanzha’ reaches 75.5% [8], higher than those of other *Crataegus* accessions, whose true seeds are usually less than 60% [10], [11].

The pyrene, usually called stone for *Prunus* species, is formed through lignification of the fruit endocarps layer, a feature of a broader class of plants [12]. Ryugo (1963) first recognized that peach stones contained lignin in the early 1960s [13]. Lignin is a compound unique to plants. Over the years, most enzymes in the lignin biosynthetic pathway and a number of potential regulatory steps in the pathway have been identified [14]. Lignin is formed from the phenylpropanoid pathway, the end products of which are coniferyl and sinapyl alcohols. These lignin monomers serve as the basis of lignification, a process lignin polymer is formed via oxidative processes guided by peroxidases and laccases [12]. Endocarp lignification in *Arabidopsis* has been well studied in relation to dehiscence [15], but the mechanism of pyrene hardening has only been investigated to a limited extent. Some enzymes in the composition and formation of stone or pyrene have been examined [16], [17], and the molecular basis for stone formation during early peach fruit development was investigated with microarrays [12].

RNA-Seq is a recently developed high-throughput sequencing method that produces millions of short cDNA reads [18]. The reads are aligned to a reference genome or reference transcripts, or are *de novo* assembled without the genomic sequence information to produce a genome-scale transcription map consisting of both transcript structure and gene expression levels [19]. RNA-Seq is a powerful and accurate tool for quantifying gene expression levels, widely regarded as more superior than the microarray-based methods [20]. Specifically, RNA-Seq can reveal novel transcribed regions, splicing isoforms, single nucleotide polymorphisms (SNPs), simple sequence repeats (SSRs), and the precise location of transcription boundaries [21]. RNA-Seq can be used in transcriptome profiling of species with no genome sequencing data.

In this work, we present a *de novo* assembly of the fruit transcriptome of *C. pinnatifida* using Illumina-based RNA-seq data. Differential gene expression between the soft-endocarp variety and the common hard endocarp variety was investigated to reveal differential regulation of key pathways.

## Results

### Temporal Pattern of Lignin Deposition in the Hawthorn Endocarp

**Figure 2:**
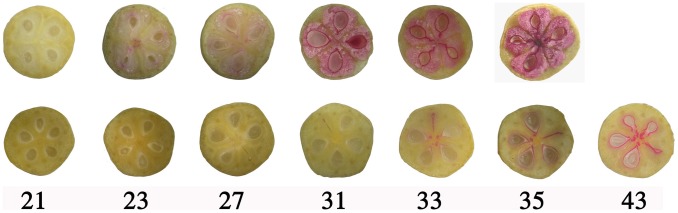
Progression of lignin deposition in developing hard-endocarp hawthorn fruit (top row) and soft-endocarp hawthorn (bottom row). Cross-sectioned fruits were stained with phloroglucinol-HCl for lignin (red colour). Numbers indicate DAB.

In order to identify the critical pyrene developmental times of hard-and soft-endocarp hawthorns, the developmental time of lignin deposition was studied by staining lignin with phloroglucinol-HCl solution. Lignin deposition was first detected in the region covering seeds of fruits of H8 (hard endocarp accession) at 23 DAB. The H8 endocarps became substantially harden by 35 DAB, after which they could not be cut with a knife. No staining was observed in tissues other than the endocarp except a few scattered vascular strands. In contrast, S7 (soft endocarp accession), did not show any lignin deposition until 31 DAB, and the endocarps were very thin even at 43 DAB (Figure 2).

### *De novo* Assembly and Assessment of the Illumina ESTs

For RNA-Seq analysis, two cDNA libraries, H8 and S7, were made from 23 DAB fruits of hawthorn. After removing low-quality reads and trimming adapter sequences, 11,538,395 and 14, 659,624 reads (76-bp in size) were obtained for H8 and S7, respectively, encompassing 2,140,589,552 and 2,743,145,672 total nucleotides (nt) respectively (Table 1). *De novo* assembly was carried out by Trinity, an assembly software to build the reference-free full-length transcription, designed specifically for high-throughput RNA sequencing [22]. The mean length of contigs was about 130 bp, and the numbers of >200 bp contigs were 460,119 for H8 and 515,118 for S7 (Table 1). The transcripts were constructed by the Butterfly program of Trinity. We obtained 54,662 transcripts for H8, and 62,653 transcripts for S7, with average lengths of 756 and 846 bp, respectively (Table 1).

**Table 1:** Summary of RNA-seq and de novo assembly of *C. pinnatifida* unigenes.

Sequences	H8	S7
Total nucleotides	2,140,589,552	2,743,145,672
Number of clean reads	11,538,395	14,659,624
Number of >200 bp contigs	460,119	515,118
Mean length of contigs (bp)	130	131
Number of >200 bp transcripts	54,662	62,653
Mean length of transcripts (bp)	756	846
N50 length of transcripts (bp)	1,237	1,421
Number of Unigenes	39,663	41,723
Mean length of Unigenes (bp)	656	703
N50 length of Unigenes (bp)	1,083	1,227

These transcripts were assembled into 39,663 putative unigenes for H8 and 41,723 putative unigenes for S7, with the mean length of 656 and 703 bp, respectively (Table 1). After combining the unigene data from H8 and S7, the unigene database of the hawthorn was established and contained 52,673 putative unigenes. The sequence information of all unigenes has been deposited in the National Center for Biotechnology Information (NCBI) under the accession GALU00000000. The mean length of the putative unigenes was 674 bp. Among all putative unigenes of hawthorn, 10,744 (7461+3283) putative unigenes have lengths of more than 1,000 bp, representing 20.4% (10,744/52,673) of total putative unigenes (Table 2). The size distribution of the assembly unigenes is shown in Figure S1.

**Table 2:** Length of *C. pinnatifida* unigenes.

Length of unigene (bp)	No. of unigenes	Percentage (%)
200–300	20,637	39.2
300–500	12,767	24.2
500–1,000	8,525	16.2
1,000–2,000	7,461	14.2
2000+	3,283	6.2
Total	52,673	

The putative unigenes with abundant transcripts (RPKM>100) and large expression differences between H8 and S7 [log_2_(S7/H8) >5 or <−5], were chosen for further analysis regarding EST quality. Among the 63 putative unigenes analyzed, 52 (82.5%) have complete CDS. Both hawthorn and apple are pome fruits. A high-quality draft genome sequence of the domesticated apple (*Malus×domestica*) was finished [23], and predicted CDS sequences of apple were available on website (http://genomics.research.iasma.it/). We compared the length and identity of CDS between hawthorn and apple. Among the 52 hawthorn putative unigenes with complete CDS, 38 have a homolog in the apple genome. The identity of CDS between hawthorn and apple gene ranges from 65.63% to 99.09%, with the mean of 90.80% (Table S1).

We detected SSRs among >1,000 bp putative unigene sequences using the MISA program (Table S2). SSRs were identified from 3,174 putative unigene sequences, which represent about 29.5% (3,174/10,744) of the analyzed unigene sequences. SSRs with mono-, di-, tri-, tetra-, penta- and hexanucleotide repeats composed about 31.3%, 39.5%, 21.3%, 0.9%, 0.1% and 0.1% of the SSRs, respectively. And 212 putative unigene sequences with the compound SSR were identified. These SSR will serve as the basis for future marker development.

### Functional Annotation and Characterization of Unigenes

**Figure 3:**
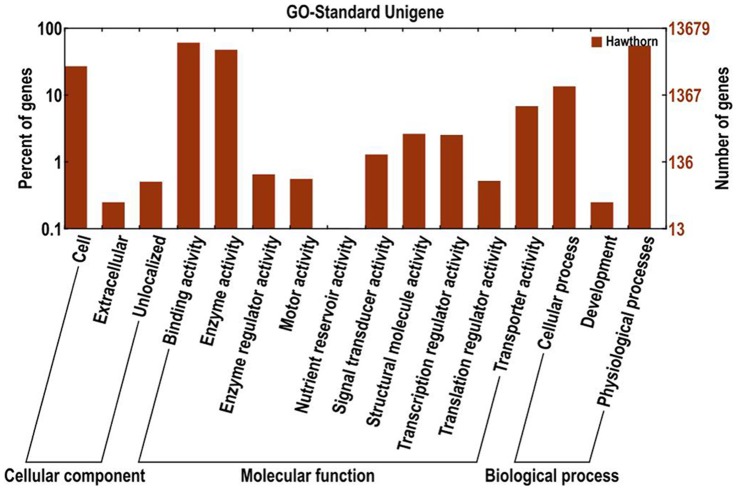
Histogram of GO classifications of assembled *C. pinnatifida* unigenes. Results are summarized in three main GO categories: cellular component, molecular function and biological process.

The entire unigene sets were annotated on the basis of similarities to known or putative sequences in the public databases. Among the 52,673 high-quality unique sequences, 35,979 (68.3%) had at least one significant match to an existing gene model in BLAST searches (Table 3). Based on sequence homology, 13,922 putative unigenes of *C. pinnatifida* were categorized into 16 functional groups, belonging to three main GO ontologies: cellular component, molecular function and biological process (Figure 3). The results show that a high percentage of genes from categories of “physiological process”, “binding activity”, “enzyme activity”, “cell” and “cellular process”, and with only a few genes related to “extracellular”, “development” and “nutrient reservoir activity”.

**Table 3:** Summary of annotations of assembled hawthorn (*C. pinnatifida*) unigenes.

Category	Number of unigenes ≥300 bp	Number of Unigenes ≥1,000 bp	Number of total annotated unigenes	Percentage (%)^c^
COG_Annotation	3,141	4,746	9,323	17.7
GO_Annotation	4,819	7,217	13,922	26.4
Kegg_Annotation	14,061	10,597	32,466	61.6
Swiss-Prot_Annotation	9,103	9,251	22,891	43.5
TrEMBL_Annotation	13,185	10,451	30,465	57.8
InterProScan_Annotation	6,947	9,005	18,719	35.5
Nr^a^_Annotation	14,270	10,602	32,757	62.2
Nt^b^_Annotation	11,880	10,208	28,413	53.9
All_Annotation	15,569	10,660	35,979	68.3

a Nr = NCBI non-redundant sequence database.

b Nt = NCBI nucleotide sequence database.

c Proportion of the 52,673 assembled unigenes.

The 100 most abundant unigenes were analyzed (Table S3). Sixty-one unigenes were highly expressed both in H8 and S7. Among the 39 unigenes that were highly expressed in hard-endocarp hawthorn (H8) but not in soft-endocarp hawthorn (S7), some were lignin biosynthetic genes, including CCoA-OMT (caffeoyl-CoA O-methyltransferase) and CAD (cinnamyl-alcohol dehydrogenase) genes. By contrast, some genes encoding flavonoid biosynthetic enzymes, incluing CHS (chalcone synthase), CHI (chalcone isomerase) and ANR (anthocyanidin reductase) were highly expressed in S7 but not in H8.

### Transcript Differences between Hard and Soft Endocarps

We used the general chi-squared test with a random sampling model in the IDEG6 software [24] to identify genes differentially expressed in fruits between soft-endocarp hawthorn (S7) and hard endocarp control (H8). A total of 1,218 genes, represented 2.31% (1,218/52,673) total putative unigenes, were differentially expressed between H8 and S7 (twofold or more change and *p*<0.001). The detail information can be accessed through Table S4. Among the genes that were differentially expressed between the two accessions, 537 genes were up-regulated and 681 genes were down-regulated in soft-endocarp hawthorn (S7). In addition, 85.96% (1,047/1218) of the differentially expressed genes were detected in fruits of both accessions. To illustrate the differential expression of genes detected in the fruits of hard endocarp hawthorn and soft endocarp hawthorn, we assigned GO functional classes to the differentially expressed genes with putative functions. These genes were sorted into major functional categories (Figure S2).

**Figure 4:**
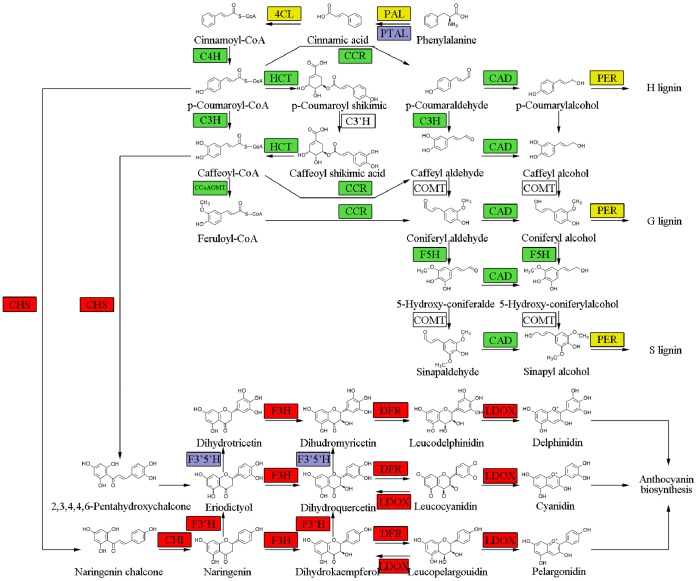
Lignin and flavonoid biosynthetic pathway genes differentially expressed in fruits between the hard-endocarp hawthorn (H8) and the soft-endocarp hawthorn (S7). Green colour means the gene was significantly down-regulated in S7, red colour means the gene was significantly up-regulated in S7, yellow colour means that the expression models of annotated unigenes were inverse, and blue colour means expression difference was not significant.

In an effort to identify key genes responsible for lignin deposition in the hawthorn endocarp, lignin biosynthetic pathway genes were identified from the 1,218 differentially expressed genes. Most lignin pathway genes were down-regulated in the fruits of soft-endocarp hawthorn (S7) compared to the fruits of control hard-endocarp hawthorn (H8), including the genes encoding C4H (cinnamate 4-hydroxylase) [EC 1.14.13.11], HCT (hydroxycinnamoyl-CoA shikimate/quinate hydroxycinnamoyl transferase) [EC 2.3.1.133], C3H (p-coumarate 3-hydroxylase) [EC 1.14.13.-], CCR (cinnamoyl-CoA reductase) [EC 1.2.1.44], CCoA-OMT [EC 2.1.1.104], F5H (ferulate-5-hydroxylase) [EC 1.14.-.-] and CAD [EC 1.1.1.195] (Figure 4; Table 4). Both PAL (phenylalanine ammonia-lyase) [EC 4.3.1.24] and 4CL (4-coumarate:CoA ligase) [EC 6.2.1.12] were annotated by two inversely expressed unigenes, i.e., 7_Unigene_BMK.17577 [log_2_(S7/H8) = 2.71] and 8_Unigene_BMK.5888 [log_2_(S7/H8) = −2.59] were annotated as PAL gene, while 7_Unigene_BMK.4691 [log_2_(S7/H8) = 3.25] and 8_Unigene_BMK.37554 [log_2_(S7/H8) = −3.29] were annotated as 4CL gene. Four unigenes were annotated as the gene encoding peroxidase [1.11.1.7]. Among them, two were down-regulated in the fruits of soft-endocarp hawthorn (S7) [8_Unigene_BMK.15024, log_2_(S7/H8) = −14.97; 7_Unigene_BMK.36531, log_2_(S7/H8) = −3.63] and two were up-regulated [7_Unigene_BMK.930, log_2_(S7/H8) = 5.51; 8_Unigene_BMK.33978, log_2_(S7/H8) = 1.12] (Table 4). The gene expression ratios for each selected lignin pathway gene were inserted into the phenylpropanoid biosynthetic pathway [KEGG map00940 (http://www.genome.jp/kegg-bin/show_pathway?map00940)], and the detail information can be accessed through the File S1. Laccases, in conjunction with peroxidase, are postulated to be involved in lignin polymerization and cell wall lignification by catalyzing the one-electron oxidation of monolignols [25]. Eleven unigenes were annotated as laccase [EC 1.10.3.2] gene, and ten were down-regulated in the fruits of soft-endocarp hawthorn (S7) with the Log_2_(S7/H8) fold value from –17.54 to –3.42 except one that was up-regulated [7_Unigene_BMK.36241, log_2_(S7/H8) = 3.94] (Table 4).

**Table 4:** Expression profiles of lignin and flavonoid biosynthesis genes in *C. pinnatifida.*

Unigene ID	Annotation	Function	log_2_(S7/H8)
7_Unigene_BMK.17577	PAL	Lignin and flavonoid biosynthesis	2.71
8_Unigene_BMK.5888	PAL	Lignin and flavonoid biosynthesis	−2.59
7_Unigene_BMK.4691	4CL	Lignin and flavonoid biosynthesis	3.25
8_Unigene_BMK.37554	4CL	Lignin and flavonoid biosynthesis	−3.29
8_Unigene_BMK.39561	C4H	Lignin and flavonoid biosynthesis	−1.38
7_Unigene_BMK.21369	C3H	Lignin and flavonoid biosynthesis	−3.66
8_Unigene_BMK.5677	C3H	Lignin and flavonoid biosynthesis	−5.06
8_Unigene_BMK.7232	HCT	Lignin biosynthesis	−4.58
8_Unigene_BMK.8160	CCR	Lignin biosynthesis	−3.06
7_Unigene_BMK.18764	CCoA-OMT	Lignin biosynthesis	−4.52
8_Unigene_BMK.16089	F5H	Lignin biosynthesis	−6.25
8_Unigene_BMK.34885	CAD	Lignin biosynthesis	−2.67
8_Unigene_BMK.15024	Peroxidase	Lignin biosynthesis	−14.97
7_Unigene_BMK.36531	Peroxidase	Lignin biosynthesis	−3.63
7_Unigene_BMK.930	Peroxidase	Lignin biosynthesis	5.51
8_Unigene_BMK.33978	Peroxidase	Lignin biosynthesis	1.12
8_Unigene_BMK.36210	Laccase	Lignin polymerization	–17.54
8_Unigene_BMK.8811	Laccase	Lignin polymerization	–3.42
7_Unigene_BMK.36241	Laccase	Lignin polymerization	3.94
8_Unigene_BMK.11701	CHS	Anthocyanin biosynthesis	3.82
7_Unigene_BMK.26121	CHI	Anthocyanin biosynthesis	3.34
7_Unigene_BMK.4912	CHI	Anthocyanin biosynthesis	3.29
8_Unigene_BMK.11985	F3′H	Anthocyanin biosynthesis	1.78
7_Unigene_BMK.39020	F3H	Anthocyanin biosynthesis	2.92
7_Unigene_BMK.26525	DFR	Anthocyanin biosynthesis	3.66
7_Unigene_BMK.37947	LAR	Anthocyanin biosynthesis	4.70
7_Unigene_BMK.41411	LDOX	Anthocyanin biosynthesis	3.45
7_Unigene_BMK.726	ANR	Anthocyanin biosynthesis	3.20

While most lignin pathway genes were down-regulated in the fruits of soft-endocarp hawthorn, almost all the flavonoid biosynthetic pathway genes were strongly up-regulated in the fruits of soft-endocarp hawthorn (S7) compared to the fruits of control hard-endocarp hawthorn (H8), including the genes encoding CHS [EC 2.3.1.74], CHI [EC 5.5.1.6], F3′H (flavonoid 3′-hydroxylase) [EC 1.14.13.21], F3H (flavanone 3-hydroxylase) [EC 1.14.11.9], DFR (dihydroflavonol 4-reductase) [EC 1.1.1.234], LAR (leucoanthocyanidin reductase) [EC 1.17.1.3], LDOX (leucoanthocyanidin dioxygenase) [EC 1.14.11.19] and ANR (anthocyanidin reductase) [EC 1.3.1.77] (Figure 4; Table 4). The gene expression ratios for each selected flavonoid pathway gene were shown in the flavonoid biosynthetic pathway [KEGG map00941 (http://www.genome.jp/kegg-bin/show_pathway?map00941)], and the detail information can be accessed through File S2.

Endocarp is mainly composed of lignin and polysaccharides such as cellulose and hemicelluloses. The expressions of some sugar metabolism pathway genes are significantly changed between soft-endocarp hawthorn (S7) and hard-endocarp hawthorn (H8). UDP-glucose is the substrate of both cellulose and pectin synthesis, the expressions of genes encoding enzymes to catalyze UDP-glucose synthesis and metabolism, were down-regulated in the fruits of soft-endocarp hawthorn (S7), including phosphoglucomutase [EC 5.4.2.2], UGPase (UDP–glucose pyrophosphorylase, also named UTP-glucose-1-phosphate uridylyltransferase) [EC 2.7.7.9], UGDH (UDP glucose 6-dehydrogenase) [EC 1.1.1.22] and sucrose synthase [EC 2.4.1.13]. The expressions of alpha-1,4-galacturonosyltransferase [EC 2.4.1.43], beta-fructofuranosidase [EC 3.2.1.26] and fructokinase [EC 2.7.1.4] genes were also down-regulated in the fruits of S7, while the expressions of beta-glucosidase [EC 3.2.1.21], pectinesterase [EC 3.1.1.11] and UDP-glucuronate 4-epimerase [EC 5.1.3.6] genes were up-regulated in the fruits of S7. The gene expression ratios for each selected sugar metabolism pathway gene were shown into the starch and sucrose metabolism pathway [KEGG map00500 (http://www.genome.jp/kegg-bin/show_pathway?map00500)], and the detail information can be accessed through the File S3.

To identify regulatory factors that potentially control lignin and flavonoid biosynthesis, candidate transcription factors were chosen from the transcriptome data. We initially targeted MYB proteins which are key factors in the regulation of primary and secondary metabolism [26]. Fifteen candidate MYB genes were identified from the 1,218 differentially expressed genes, among them, 12 MYB genes were down-regulated in the fruits of soft-endocarp hawthorn (S7), while 3 MYB genes were up-regulated (Table S5). The 8_Unigene_BMK.10344 and 8_Unigene_BMK.9503 were annotated as *MYB46* genes, which appears to have a more direct role in the control of secondary wall formation [15]. The 8_Unigene_BMK.10344 was strongly down-regulated in the fruits of soft-endocarp hawthorn (S7) [log_2_(S7/H8) = –15.36]. Four MYB genes were annotated as *R2R3-MYB* genes, and among them, 7_Unigene_BMK.1607 is clustered with apple *MYB10*, which is known to control anthocyanin biosynthesis [27]. The 7_Unigene_BMK.1607 was strongly up-regulated in the fruits of S7 [log_2_(S7/H8) = 14.05]. Secondly we targeted NAC transcription factors, which are known to control secondary wall formation in woody tissues [28]. Four candidate NAC genes were identified from the 1,218 differentially expressed genes, and all of them were strongly down-regulated in the fruits of soft-endocarp hawthorn (S7) compared to the fruits of control hard-endocarp hawthorn (H8), including 8_Unigene_BMK.34172 [log_2_(S7/H8) = −14.67], 8_Unigene_BMK.37276 [log_2_(S7/H8) = −6.31], 7_Unigene_BMK.19150 [log_2_(S7/H8) = −3.98] and 8_Unigene_BMK.30887 [log_2_(S7/H8) = −3.00].

**Figure 5:**
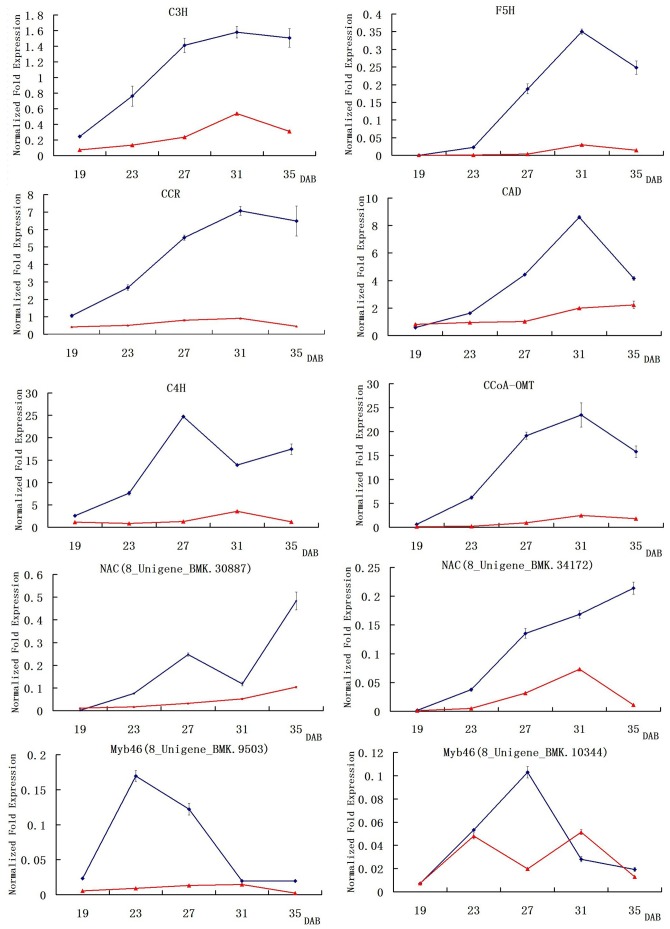
The expression levels of 6 lignin pathway genes (C3H, C4H, CAD, CCoA-OMT, CCR and F5H) and Myb46 and NAC genes using qRT-PCR. Blue lines mean the hard-endocarp hawthorn (H8), red lines mean the soft-endocarp hawthorn (S7). Y-axis represents normalized fold expression, X-axis is DAB (Days after bloom).

To confirm the results of Illumina RNA-Seq analysis, the expression levels of 6 lignin pathway genes (*C3H, C4H, CAD, CCoA-OMT, CCR* and *F5H*) and Myb46 and NAC genes were measured in H8 and S7 at 19, 23, 27, 31 and 35 DAB by qRT-PCR. The qRT-PCR data for all the tested genes (Figure 5) were consistent with those obtained by Illumina RNA-Seq analysis.

## Discussion

With the application of massively parallel sequencing technologies, transcriptome information is becoming generally abundant not only for model organisms on which international research efforts and funding are concentrated, but also for non-model organisms. In this study, we generated 2.14 Gb and 2.74 Gb of raw sequence data by Illumina sequencing of hard-endocarp hawthorn and soft-endocarp hawthorn fruits, corresponding to 39,663 and 41,723 putative unigenes, respectively. For Illumina RNA-Seq, the mean size of assembled of unigenes was several hundred base pairs [18], [21], [29], [30]. The mean size of assembled of hawthorn’s putative unigenes was 674 bp, and among the total 52,673 putative unigenes, 10,744 putative unigenes were ≥1,000 bp in length (Table S2). For unigenes with abundant transcripts and large expression differences between H8 and S7, 82.5% have complete CDS. These suggest that we have obtained high quality EST data for hawthorn, facilitating future gene cloning from *Crataegus* genus for which no EST data are available in NCBI now.

Genome-wide transcriptome analysis between the soft-endocarp hawthorn and the hard-endocarp hawthorn using RNA-Seq technology revealed significant down-regulation of a number of lignin pathway genes (Table 4) concurrent with the reduction of lignin deposition and soft-endocarp forming. Down-regulation of C4H, HCT, C3H, CCR or CcoA-OMT was previously reported to cause reduction of total lignin in transgenic alfalfa, *Arabidopsis,* and poplar [31]–[35]. The Log_2_(S7/S8) fold value of unigene annotated as F5H was lowest (–6.25) (Table 4). F5H, a cytochrome P450-dependent monooxygenasea [36], [37], is a key enzyme that catalyzes the hydroxylation of ferulic acid, coniferaldehyde, and coniferyl alcohol, leading to sinapic acid and syringyl lignin (S lignin) biosynthesis [38]. F5H affects partitioning between two major traditional monolignols, coniferyl and sinapyl alcohols [39]. The *Arabidopsis* mutant deficient in F5H contained only traces of S lignin [40], while the F5H overexpressed transgenic plants displayed substantially more S lignin and was consequentially severely depleted in coniferyl alcohol-derived G lignin [39], [40]. The down-regulation or overexpression of F5H by transgenic approach had little effect on the total lignin [34], [41]. However, there was a substantial change in the distribution between acid-soluble and insoluble lignin fractions, with the transgenic poplar trees displaying elevated levels of acid-soluble lignin and reduced levels of acid-insoluble lignin in comparison to wild-type [39]. The function of hawthorn F5H gene will be elucidated by future overexpression and silencing in hawthorn.

In vascular plants, the phenylpropanoid pathway is responsible for the biosynthesis of a variety of metabolites, including lignin and flavonoids [42]. Lignin and flavonoid biosynthesis are competitive since they presumably draw on the same precursors and diverge at the common intermediate *p*-coumaroyl CoA. For peach, during times of peak lignin deposition, genes in the lignin biosynthesis pathway were strongly induced while flavonoid pathway genes were repressed [12]. Conversely, high flavonoid gene expression was correlated with lower expression of lignin biosynthesis genes. Our gene expression data confirm the competing effect of lignin and flavonoid biosynthetic pathways. Hawthorn fruits are a rich source of flavonoids [43]. Flavonoid consumption has been documented to be negatively associated with cardiovascular conditions, so hawthorn has been used as an alternative therapy for a variety of heart disease mortality [1]. Whether the soft-endocarp hawthorn accumulates more flavonoids than common hard-endocarp hawthorns is unknown and should be tested in the future. Our work provides candidate genes for improving nutritional value of hawthorn.

The phenylpropanoid pathway genes are developmentally regulated by various classes of *cis*-acting elements [44] and *trans*-acting transcription factors [15], [45]. Putative AC elements are *cis*-acting elements found in the majority promoters of phenylpropanoid biosynthetic genes, including *PAL*, *4CL*, *C3H*, *CCoAOMT*, *CCR* and *CAD* [46], and it has been proposed that coordinated expression of these genes is regulated by MYBs which bind the AC elements. Thus, regulation by lignin activators is global rather than specific for certain pathway genes [15]. In this study, 15 candidate MYB genes were identified from the differentially expressed genes, and 12 of them were down-regulated in the fruits of soft-endocarp hawthorn. It indicates that the lignin deposition in hawthorn endocarp is regulated by MYB transcription factor. MYBs are not the only transcription factors that can regulate lignin pathways. Some MYBs known to be involved in controlling lignin deposition and secondary wall formation are activated by NAC transcription factors in *Arabidopsis* [47]. For example, MYB46 and MYB83 are the direct targets of SND1, a NAC domain transcription factor [48]. In this study, four candidate NAC genes were identified from the differentially expressed genes, and all of them were strongly down-regulated in the fruits of soft-endocarp hawthorn. So, NAC domain transcription factors are important candidate regulatory genes for lignin biosynthesis in endocarp of hawthorn.

This study reports the first application of RNA-Seq technology for transcriptome studies in hawthorn, a non-model species with few genomic resources. Our results demonstrate that RNA-Seq can be successfully used to obtain high quality EST data of hawthorn and to identify differentially expressed genes between the soft-endocarp hawthorn and the hard-endocarp hawthorn. This work revealed significant down-regulation of a number of lignin biosynthetic genes and up-regulation of almost all flavonoid biosynthetic genes, concomitant with the reduction of lignin deposition and soft-endocarp forming. In addition, we have identified MYB and NAC transcription factors that potentially control lignin and flavonoid biosynthesis. The function of these differentially expressed genes, in particular the MYB and NAC factors, between the soft-endocarp and the hard-endocarp hawthorn will be elucidated in the future.

## Materials and Methods

### Plant Material

The trees of *Crataegus pinnatifida* accessions H8 (hard-endocarp hawthorn) and S7 (soft-endocarp hawthorn) were maintained in the National Hawthorn Germplasm Repository at Shenyang. Bloom time was noted when 50% of flowers had opened. The fruits for analysis of lignin deposition were collected once every two days from 19 to 43 days after bloom (DAB). At each collection time, 16 fruits were collected from each accession. Ten of these were frozen in liquid N_2_ for future RNA extraction. The remaining six were transversely cut into sections and immediately stained with phlorogucinol solution [2% phloroglucinol, 85% ethanol (v/v)] for 2 min, drained and exposed to 85% HCl for 2 min. The fruit sections were then rinsed in 95% ethanol (v/v) and photographed. The ripen fruits were collected at 118 DAB (Figure 1).

### RNA Extraction and Quality Determination

Total RNA were isolated using the modified CTAB method performed as Chang et al. [49], and the RNA samples were treated with DNase I (TaKaRa, Japan) for 4 h. The integrity of the RNA samples was examined with an Agilent 2100 Bioanalyzer (Agilent Technologies, Palo Alto, USA).

### cDNA Library Preparation and Illumina Sequencing

cDNA library preparation and sequencing reactions were conducted in the Biomarker Technology Company, Beijing, China. The paired-end library preparation and sequencing were performed following standard Illumina methods using the DNA sample kit (#FC-102–1002, Illumina). The cDNA library was sequenced on the on the Illumina sequencing platform (HiSeq™ 2000).

### *De Novo* Assembly

Reads from each library were assembled separately. The trimming adapter sequences were removed and low-quality reads (less than 13 bp or reads with unknown nucleotides larger than 5%) were filtered with the software developed by the Biomarker Technology Company. The Trinity method [22] was used for *de novo* assembly of Illumina reads of hawthorn. Trinity consists of three software modules: Inchworm, Chrysalis and Butterfly, applied sequentially to process large volumes of RNA-Seq reads. In the first step in Trinity, reads are assembled into the contigs by Inchworm program. The minimally overlapping contigs were clustered into sets of connected components by Chrysalis program, and then the transcripts were constructed by the Butterfly program [22]. In this study, only one k-mer length (25-mer) was chosen in Trinity, using the follow parameters: seqType fq, group_pairs_distance = 150 and other default parameters. Finally, the transcripts were clustered by similarity of correct match length beyond the 80% of longer transcript or 90% of shorter transcript used multiple sequence alignment tool BLAT [50]. The longest transcript of each cluster was taken as the unigene. The Illumina data set has been deposited in NCBI Sequence Read Archive (SRA) under accession number SRX305204.

### SSRs Detection

SSRs were detected among the unigenes with length >1,000 bp using the software MISA (MIcroSAtellite; http://pgrc.ipk-gatersleben.de/misa) [51]. Total 7 types of SSRs were investigated, including mono-, di-, tri-, tetra-, penta- and hexanucleotide repeats, and the compound SSR (the sequence contains two adjacent distinct SSRs separated by none to any number of base pairs).

### CDS Analysis

The coding sequence (CDS) in unigene was predicted by EMBOSS getorf program. The complete CDS sequences of hawthorn unigenes, with abundant transcripts (RPKM>100) and large expression differences between H8 and S7 [log_2_(S7/H8)>5 or <−5], were chosen to compare with the predicted CDS sequences of apple (*Malus*×*domestica*) (http://genomics.research.iasma.it/), and the identity of CDS between the hawthorn and apple gene was analyzed with DNAMAN software (version 5.2.2).

### Functional Annotation

We annotated unigenes based on a set of sequential BLAST searches [52] designed to find the most descriptive annotation for each sequence. The assembled unigenes were compared with sequences in NCBI non-redundant (Nr) protein and nucleotide (Nt) databases (http://www.ncbi.nlm.nih.gov), the Swiss-Prot protein database (http://www.expasy.ch/sprot), the Kyoto Encyclopedia of Genes and Genomes (KEGG) pathway database (http://www.genome.jp/kegg), the Cluster of Orthologous Groups (COG) database (http://www.ncbi.nlm.nih.gov/COG), the Translated EMBL Nucleotide Sequence database (TrEMBL) (http://www.uniprot.org/) and InterPro database (http://www.ebi.ac.uk/interpro/). The Blast2GO program [53] was used to obtain GO annotation of the unigenes. The WEGO software (http://wego.genomics.org.cn/cgi-bin/wego/index.pl) was then used to perform GO functional classification of all unigenes to view the distribution of gene functions.

### Digital Gene Expression Analysis

Gene expression levels were measured in the RNA-Seq analysis as reads per kilobase of exon model per million mapped reads (RPKM) [54]. The IDEG6 software [24] was used to identify differentially expressed genes in pair-wise comparison, and the results of all statistical tests were corrected for multiple testing with the Benjamini–Hochberg false discovery rate (FDR<0.01). Sequences were deemed to be significantly differentially expressed if the adjusted *P* value obtained by this method was <0.001 and there was at least a twofold change (>1 or <−1 in log 2 ratio value) in RPKM between two libraries.

### Quantitative RT-PCR (qRT-PCR) Analysis

The cDNA was synthesized from total RNA using Reverse Transcriptase XL (AMV) (TaKaRa, Japan) in a 20 µL reaction system. The reverse transcription reaction mixture contained 5 µL total RNA (1 µg), 1 µL 10 mM of each dNTP, 1 µL of random primer (9 mer) (50 µM), 1 µL oligo d(T)_18_ primer (50 µM) (TaKaRa, Japan) and 6 µL DEPC water. The mixture were incubated at 65°C for 5 min and cooled on ice for 5 min, then 4 µL 5×Reverse Transcriptase buffer, 1 µL RNasin (TaKaRa, Japan) and 1 µL AMV (5 U) were added. The mixture was incubated at 37°C for 2.5 h. The enzyme was inactivated by incubating at 72°C for 15 min. qPCR was carried out on the iQ5 Real Time PCR Detection System (BioRad, USA) with RealMasterMix SYBR Green (TIANGEN, China). Primers used in qPCR for validation of differentially expressed genes were shown in Table S6. Each gene was analyzed in triplicate, after which the average threshold cycle (CT) was calculated per sample, and an endogenous *ACTIN* gene was used for normalization. Relative fold changes in genes expression were calculated using the comparative Ct (2^−ΔΔCt^) method.

## Supporting Information

Figure S1 **The size distribution of the** ***C. pinnatifida*** **unigenes.**
(EPS)

Figure S2 **Functional categories of 1,218 differentially expressed unigenes between the hard-endocarp hawthorn (H8) and the hard-endocarp hawthorn (S7).**
(EPS)

Table S1 **EST quality of** ***C. pinnatifida*** **unigenes.**
(XLS)

Table S2 **Detail information of SSRs in** ***C. pinnatifida*** **unigenes.**
(XLS)

Table S3 **The 100 most abundant unigenes in the two hawthorns sample sets.**
(XLS)

Table S4 **All the differentially expressed unigenes between the hard-endocarp hawthorn (H8) and the soft-endocarp hawthorn (S7).**
(XLS)

Table S5 **Candidate MYB genes identified from** ***C. pinnatifida.***
(XLS)

Table S6 **Primers used to perform qPCR of lignin biosynthesis and regulate genes.**
(XLS)

File S1 **Phenylpropanoid biosynthetic pathway (KEGG map00940) genes differentially expressed in fruits between the hard-endocarp hawthorn (H8) and the soft-endocarp hawthorn (S7).**
(RAR)

File S2 **Flavonoid biosynthetic pathway (KEGG map00941) genes differentially expressed in fruits between the hard-endocarp hawthorn (H8) and the soft-endocarp hawthorn (S7).**
(RAR)

File S3 **Starch and sucrose metabolism pathway (KEGG map00500) genes differentially expressed in fruits between the hard-endocarp hawthorn (H8) and the soft-endocarp hawthorn (S7).**
(RAR)
